# Clinical optoacoustic imaging combined with ultrasound for coregistered functional and anatomical mapping of breast tumors

**DOI:** 10.1016/j.pacs.2018.08.003

**Published:** 2018-08-31

**Authors:** A.A. Oraevsky, B. Clingman, J. Zalev, A.T. Stavros, W.T. Yang, J.R. Parikh

**Affiliations:** a TomoWave Laboratories, Houston, TX, United States; b Seno Medical Instruments, San Antonio, TX, United States; c Department of Physics, Ryerson University, Toronto, Canada; d Department of Radiology, University of Texas MD Anderson Cancer Center, Houston, TX, United States

**Keywords:** Optoacoustic, Photoacoustic, Ultrasound, Functional-anatomical imaging, Breast cancer, Diagnostics, Dual modality

## Abstract

Optoacoustic imaging, based on the differences in optical contrast of blood hemoglobin and oxyhemoglobin, is uniquely suited for the detection of breast vasculature and tumor microvasculature with the inherent capability to differentiate hypoxic from the normally oxygenated tissue. We describe technological details of the clinical ultrasound (US) system with optoacoustic (OA) imaging capabilities developed specifically for diagnostic imaging of breast cancer. The combined OA/US system provides co-registered and fused images of breast morphology based upon gray scale US with the functional parameters of total hemoglobin and blood oxygen saturation in the tumor angiogenesis related microvasculature based upon OA images. The system component that enabled clinical utility of functional OA imaging is the hand-held probe that utilizes a linear array of ultrasonic transducers sensitive within an ultrawide-band of acoustic frequencies from 0.1 MHz to 12 MHz when loaded to the high-impedance input of the low-noise analog preamplifier. The fiberoptic light delivery system integrated into a dual modality probe through a patented design allowed acquisition of OA images while minimizing typical artefacts associated with pulsed laser illumination of skin and the probe components in the US detection path. We report technical advances of the OA/US imaging system that enabled its demonstrated clinical viability. The prototype system performance was validated in well-defined tissue phantoms. Then a commercial prototype system named Imagio™ was produced and tested in a multicenter clinical trial termed PIONEER. We present examples of clinical images which demonstrate that the spatio-temporal co-registration of functional and anatomical images permit radiological assessment of the vascular pattern around tumors, microvascular density of tumors as well as the relative values of the total hemoglobin [tHb] and blood oxygen saturation [sO2] in tumors relative to adjacent normal breast tissues. The co-registration technology enables increased accuracy of radiologist assessment of malignancy by confirming, upgrading and/or downgrading US categorization of breast tumors according to Breast Imaging Reporting And Data System (BI-RADS). Microscopic histologic examinations on the biopsied tissue of the imaged tumors served as a gold standard in verifying the functional and anatomic interpretations of the OA/US image feature analysis.

## 1 Introduction

Breast cancer is the most common type of cancer in women and the second leading cause of death due to cancer among women in the United States. The American Cancer Society (ACS) estimates that 40,290 women will die from breast cancer in the United States [1,2]. Mammography is the most common imaging modality currently used for population screening and the early detection of breast cancer. While the sensitivity of screening mammography is about 87.0% in women with almost entirely fatty breasts, higher breast density and heterogeneity of breast parenchymal density limits the sensitivity of mammography to about 29–63% [2,3]. The average positive predictive value (PPV1) of mammography is only 50–60% [4]. In balancing the detection error rates against the potential harm of cumulative ionizing radiation, the ACS guidelines no longer recommend routine screening (x-ray) mammograms for patients younger than 45 years old with an average risk of breast cancer [2]. The ACS guidelines do still recommend bi-annual screening mammograms for women beyond 45 years old [2].

Diagnostic US is widely performed in the workup of abnormal mammography findings. Advantages of US include its safety, convenience and capability to visualize tumors with video rate display that are radiologically occult, lack of ionizing radiation, and relatively low cost. Targeted diagnostic breast ultrasound helps in classifying breast cancer with excellent sensitivity, but suffers from low specificity. Ultrasound diagnosis of breast cancer has been primarily based on the lesion morphology (shape characteristics and ultrasound properties). Many malignant breast masses are too small to present sufficiently distinctive features on conventional ultrasound. Thus, the positive predictive value of the diagnostic ultrasound imaging after mammography and diagnostic ultrasound in biopsied masses (PPV3) is under 30% [5]. When the ultrasound results are abnormal or indeterminate, the radiologist typically recommends that a biopsy is performed of the lesion. Because of the low PPV3, over 70% of breast biopsies are performed on benign lesions. This high false positive biopsy rate leads to about 1.6 Million unnecessary biopsy procedures with associated medical expenses of over $3Billion, patient and family anxiety, and patient discomfort from the procedure [6]. The false positive rate of supplemental screening breast ultrasound has been even higher than that of diagnostic breast ultrasound (as low as 8% PPV3 in the ACRIN 6666 trial), and has been a major obstacle to the adoption of supplemental breast ultrasound screening in women with radiologically dense and heterogeneous breast tissue [7].

### 1.1 Background and significance

We present a newly developed technology of combined optoacoustic plus ultrasound (OA/US) imaging, integrated in the Imagio™ breast imaging system, specifically designed for imaging of breast and diagnosis of breast masses. This technology provides a two-fold enhancement of the overall diagnostic accuracy, combining specificity from functional imaging with molecular specificity of hemoglobin and oxyhemoglobin with the 95-plus% sensitivity of breast ultrasound anatomical imaging [8]. We describe the OA/US system technical parameters and the design methods that enabled these advanced specifications. The design of laser, fiberoptic components, and an OA/US handheld probe enable generation of OA images while retaining all the advantages of breast ultrasound, including interactive real-time imaging, providing high contrast, high resolution images with visualization of breast and tumor morphology. We also discuss our signal processing, image reconstruction and post-processing to achieve precise co-registration and overlay of OA images with conventional ultrasound images to visualize hemoglobin distribution and blood oxygen saturation in the context of breast morphology. The system was validated in well-characterized breast tissue mimicking phantoms. Finally, we present examples of clinical images that demonstrate the clinical value of Imagio™ as a diagnostic imaging modality, with the potential to decrease false-positive mammography findings.

### 1.2 Fundamentals of optoacoustic imaging

For clinical radiologists, “seeing is believing”. Therefore, optical imaging technologies are naturally suited for medical applications. However, due to optical scattering, pure optical modalities that illuminate and sense light are not able to achieve adequate depth penetration and spatial resolution in the thickness of breast tissue necessary for complete evaluation of normal breast tissue [9]. Optoacoustic (photoacoustic) imaging was proposed to alleviate the problem of strong optical scattering within tissues [10,11]. This fusion of optical imaging and acoustic imaging uses the most compelling properties of light (high and spectrally selective optical contrast of molecules) and sound (high spatial and temporal resolution enabled by ultrasound propagating in tissues with relatively low attenuation to greater depths than can be achieved by optical imaging alone). Optoacoustic imaging is a method of deeper tissue visualization based on time-resolved detection of acoustic pressure profiles induced in tissue through absorption of near-infrared laser pulses with the pulse duration shorter than the time it takes for the optically generated ultrasound to propagate with the speed of sound through a voxel of tissue to be resolved on the image [12]. Out of numerous molecules that compose human tissue, five chromophores: hemoglobin, oxyhemoglobin, lipids, melanin and water considerably absorb deeply penetrating near-infrared (NIR) light [13]. The absorbed optical energy is converted into heat, causing a transient thermoelastic expansion that generates OA waves from the voxels that absorb NIR light stronger than the background tissue. These OA waves can be detected by an array of ultrasonic transducers and the detected and digitized signals can be used to reconstruct images using tomography algorithms [14]. If the laser wavelength matches (or close to) the peak optical absorption of one of the five dominant NIR chromophores, then the corresponding OA image can visualize tissues abundant with that specific molecule: hemoglobin in veins, oxyhemoglobin in arteries, lipids in nerves, melanin in skin and water in aqueous tissues. Since blood is the most important in supporting normal functioning of live tissues with oxygen and energy, OA images of blood distribution and its oxygen saturation have the highest medical relevance. OA imaging of the total hemoglobin [tHb] and blood oxygen saturation [sO2] represents functional imaging technology rather than tissue morphology. Due to relatively smooth distribution of diffuse blood in tissues, OA images lack vivid anatomical context with the exception of circulation (vasculature and microvasculature).

### 1.3 Dual modality systems

In the early years of development, OA imaging researchers realized that B-mode gray scale ultrasound imaging based on contrast provided by the acoustic impedance is complementary to the nature of medical information provided by the functional optoacoustic images [15,16]. The dual modality has the merit of images based on two different contrast mechanisms, functional optical and anatomical ultrasound, to be co-registered and temporally interleaved in real time, which in turn enhances each technology and can achieve greater clinical performance.

Furthermore, combining the two systems in one modality is acceptable to radiologists because they can readily adapt and associate functional information with morphology provided by co-registration of the optoacoustic and ultrasound images. With this understanding, a number of groups developed optoacoustic ultrasonic dual modality systems based on commercial ultrasound machines and commercial pulsed lasers (see, for example [[17], [18], [19], [20], [21]]). While these dual modalities take some advantage of the optical contrast in live tissues in addition to the acoustic impedance contrast of ultrasound imaging, all such system have one significant limitation. Medical ultrasound machines use relatively narrow band ultrasound transducers (central frequency ± 40%) that emit reverberating train of ∼3 ultrasonic pulses. These pulses are enveloped upon detection using Hilbert transform and can be used to reconstruct high-resolution images with maximum emitted frequency of 10–15 MHz. The need for such high frequencies limits penetration depth of medical ultrasound. However, the penetration depth of about 40–50 mm is sufficient for many medical imaging applications, including diagnostic imaging of breast cancer. On the other hand, the merit of optoacoustic imaging is to provide quantitative functional and molecular information from the volume of tissue structures. This type of information cannot be attained from the boundaries of blood vessels, tumors and other physiologically important tissue structures. Therefore, optoacoustic imaging requires that ultrasound detectors maintain sufficient sensitivity in the range of the lower frequencies down to 50–100 kHz. We termed this type of detectors “ultrawide-band ultrasonic transducers” [12]. Below, we describe the advanced features in the design of the optoacoustic plus ultrasound hand-held dual modality probe, the corresponding electronics hardware, and system software, which resolve prior technological challenges and enable viable clinical performance of Imagio™ for purposes of more accurate diagnosis of breast cancer.

### 1.4 Aggressive cancer as densely microvascular tissue

As elegantly described by J. Folkman, rapidly growing cancer cells require additional blood supply and gradually develop a dense microvascular network in or around tumors to support tumor growth and progression [22]. In the early stage of cancer development, the tumor depends on blood vessels in the surrounding healthy tissue and passive diffusion to support its continued growth. A tumor without its own independent supply of new blood vessels may be restricted in its growth to about 2 mm in size with a few million cells and be non-life threatening. Many of these breast tumors will remain at this *in situ* stage for years before switching into a rapid growth stage when the rate of the cell growth is much greater than the rate of apoptosis and requires new capillary blood vessels. Tumor-associated neovascularization allows the tumor cells to express their critical growth advantage. There is a constant requirement for vascular supply in cancerous tumors. Experimental and clinical evidence suggests that the process of metastasis is also angiogenesis-dependent [23]. Angiogenesis appears to be an important clinical marker for breast cancer development and, thus, has implications in tumor detection and differentiation using functional optoacoustic imaging.

### 1.5 Functional optoacoustic imaging of the breast

Supply of blood that carries oxygen and nutrients to and carbon dioxide and waste products away from tissues is necessary for normal functioning of the tissues and it is critically important for aggressive growth of malignant tumors. Using methods of diffuse optical spectroscopy and tomography, researchers have found that malignancies are characterized by a substantially higher presence of blood, with significantly lower oxygenation, than normal or benign tissue [[24], [25], [26]]. Any deviation from normal blood concentration (the total hemoglobin [tHb]) and blood oxygen saturation (hemoglobin oxygenation [sO_2_]) may be used for detection of tissue abnormalities such as hypoxia and angiogenesis of cancer. Therefore, imaging of vasculature, blood circulation and blood distribution in tissues and measurements of [tHb] and [sO_2_] by methods of functional imaging, can be used for detection of microvascular network of aggressively growing malignancies in the breast and for their differentiation from normal tissues and benign tumors [25].

When spatial distribution of the optical fluence, $F\left(\vec{r}\right)$ is known, and Gruneisen parameter, $\Gamma (\vec{r})$ of the thermoacoustic efficiency can be considered constant through the object of interest, one can obtain spatial distribution of the optical absorption coefficient, $\mu_{a}(\vec{r})$ from the optoacoustic images [27]. Since the optical absorption spectra for the molecular extinction coefficients of oxygenated hemoglobin, $\varepsilon_{HbO2}^{\lambda }$ and deoxygenated hemoglobin, $\varepsilon_{Hb}^{\lambda }$ are well known [28], one can potentially determine concentrations of [HbO_2_] and [Hb] molecules in the body. For purposes of functional biomedical diagnostics, parameters of the total hemoglobin, [tHb], and blood oxygen saturation, [sO_2_], can be determined from optoacoustic images acquired at multiple wavelengths, $\lambda_{i}$ of the optical illumination. In the simplest case when other tissue chromophores do not make noticeable contribution to the overall optical absorption, or when relative contribution of the other NIR absorbing molecules can be taken into consideration, the functional parameters of [tHb] and [sO_2_] can be measured from optoacoustic images acquired at two wavelengths, $\lambda_{1},\;\lambda_{2}$ as follows [29]:

(1) $$THb(\vec{r})=\left[Hb\right](\vec{r})+\left[HbO2\right](\vec{r})=\frac{\mu_{a}^{\lambda_{1}}\left(\varepsilon_{HbO2}^{\lambda_{2}}-\varepsilon_{Hb}^{\lambda_{2}}\right)-\mu_{a}^{\lambda_{2}}(\varepsilon_{HbO2}^{\lambda_{1}}-\varepsilon_{Hb}^{\lambda_{1}})}{\varepsilon_{Hb}^{\lambda_{1}}\varepsilon_{HbO2}^{\lambda_{2}}-\varepsilon_{Hb}^{\lambda_{2}}\varepsilon_{HbO2}^{\lambda_{1}}}$$

(2) $$SO2\left(\vec{r}\right)=\left[HbO2\right]/\{\left[Hb\right]\left(\vec{r}\right)+\left[HbO2\right]\left(\vec{r}\right)\}=\frac{\mu_{a}^{\lambda_{2}}\varepsilon_{Hb}^{\lambda_{1}}-\mu_{a}^{\lambda_{1}}\varepsilon_{Hb}^{\lambda_{2}}}{\mu_{a}^{\lambda_{1}}\left(\varepsilon_{HbO2}^{\lambda_{2}}-\varepsilon_{Hb}^{\lambda_{2}}\right)-\mu_{a}^{\lambda_{2}}(\varepsilon_{HbO2}^{\lambda_{1}}-\varepsilon_{Hb}^{\lambda_{1}})}$$

It is desirable to select the two wavelengths with inverted ratio of the optical absorption coefficients, *i.e.* $\varepsilon_{HbO2}^{\lambda_{1}}<\varepsilon_{Hb}^{\lambda_{1}}\;and\;\varepsilon_{HbO2}^{\lambda_{2}}>\varepsilon_{Hb}^{\lambda_{2}}$. It is also desirable to select the pair of wavelengths close to each other, so that changes in the optical scattering as a function of wavelength can be not too substantial, so that the distribution of the optical fluence, $\Phi (\vec{r})$, the effective optical attenuation, $\mu_{eff}\left(\vec{r}\right),$ and the resulting penetration depth can be considered similar at both wavelengths.

In the past decade, there have been a number of optoacoustic studies in microscopy [30], endoscopy [31] and tomography [32] that generated functional images *in vivo*. These and other published studies created a solid basis for our development of a system capable of visualization of relative estimates of [sO_2_(r)] and [tHb(r)] in the large tissue volumes including blood vessels and tumor microvasculature through normalization of the optical fluence distribution as a function of depth in the breast. We demonstrate below that functional imaging of tumor microvasculature and surrounding breast vasculature that is recruited to both feed and drain the tumor, together with depiction of [Hb] and [HbO2] concentrations, represents clinically relevant diagnostic information.

## 2 Materials and methods

### 2.1 Imagio system design

**Fig. 1:**
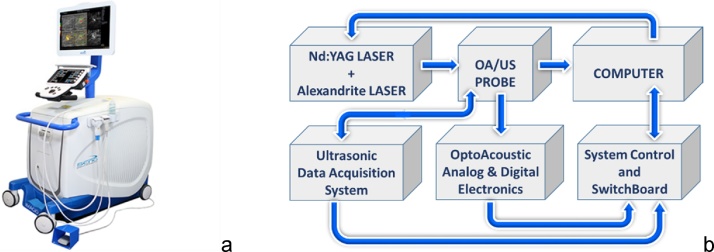
Photograph (a) and a block diagram (b) of the combined OA/US system Imagio™.

The clinical system design was based on extensive computer modeling including Monte Carlo simulations of light propagation in the breast, signal generation, propagation and detection by realistic ultrasound transducers and optimization of image reconstruction algorithms for limited view tomography [33,34], experimental development and feedback information from testing in realistic tissue phantoms [35] and pilot clinical studies [36]. The system block diagram is presented in Fig. 1.

We achieved clinical viability of OA + US technology for real-time functional anatomical mapping of the breast through (i) development of ultrawide-band ultrasonic transducer arrays and their proper loading to an ultra-low noise analog preamplifiers with high input impedance; (ii) understanding and elimination of optoacoustic image artefacts associated with hand-held probe design, which resulted in substantially increased image contrast, (iii) signal processing that inverted OA signal distortions by the detection system and (iv) image reconstruction and post-processing that computation of functional images and their accurate temporal-spatial co-registration with B-mode ultrasound images. To produce OA/US images, the Imagio system required uniquely designed subsystems: a handheld duplex OA/US probe, a laser system, OA/US reception signal processing, and OA/US image formation. Each of these subsystems is significantly different from conventional ultrasound to enable the most challenging requirement of simultaneously providing high spatial resolution (sensitivity to high ultrasonic frequencies) and high contrast of volumetric brightness of relatively large objects (sensitivity to low ultrasonic frequencies).

### 2.2 Hand-held probe design

Perhaps the most unique part of the combined optoacoustic-ultrasonic imaging system, compared to conventional ultrasound, is the specialized hand-held probe. This is also one of the most vital and challenging part of the design. We integrated a fiberoptic light delivery system with a multi-element linear array ultrasound transducer in a form that is comfortable for the clinician and patient, compact, and light, while delivering the best possible imaging performance. The probe supports sufficient imaging penetration depth in the breast, while simultaneously minimizing image “clutter” artefacts associated with proximity of optical and acoustic components.

**Fig. 2:**
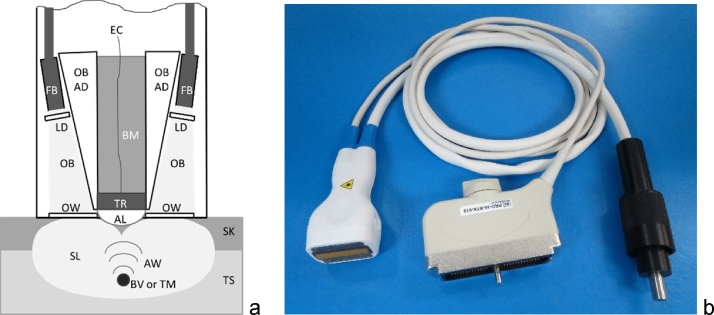
Schematic diagram (a) and a photograph (b) of the hand-held optoacoustic ultrasound probe for coregistered dual modality imaging. The following abbreviations are used: Tissue (TS), skin (SK), scattered light (SL), optical beams (OB), fiber bundles (FB), light diffusers (LD), optical windows (OW), acoustic waves (AW), blood vessels or tumors (BV or TM), scattered light (SL), acoustic lens (AL), transducers (TR), electrical cables (EC), backing material (BM).

Fig. 2 depicts a schematic diagram and a photograph of the OA/US probe used in the PIONEER clinical study. In addition to the standard functions of breast ultrasound, the optoacoustic probe is designed for the illumination of breast tissue (TS) through skin (SK) and detection of resulting transient acoustic waves. The scattered light (SL) beam is formed in the image plane of tissue by merging two optical beams (OB) emerging from the fiber bundles (FB) and passing through the light diffusers (LD) then passing through the optical windows (OW). Acoustic waves (AW) generated in blood vessels or tumors (BV or TM) by the scattered light (SL) in tissue propagate backward through acoustic lens (AL) to transducers (TR) and converted into electrical signals being transmitted through the backing material (BM) by electrical cables (EC) to the electronic analog preamplifiers.

Two of the most significant requirements for the optoacoustic probe is that (i) light cannot propagate either through the acoustic lens (AL) nor through optical block acoustic absorber on the sides of the probe, and (ii) no acoustic waves should be generated in the acoustic lens or the optical block materials [37]. To image to the depths of 40 mm needed for the clinical relevance, the probe must illuminate the breast tissue with a sufficient optical fluence that does not exceed safe levels of the incident fluence (20 mJ/cm^2^ [38]). The illumination fluence should be maximized within the imaging plane (slice), with sufficient uniformity and without sharp edges that cause acoustic artefacts. To achieve these parameters, the probe includes two optical windows, one on each side of a centrally placed ultrasound linear phased array transducer. The length of each window was selected to correspond to that of the linear array of ultrasonic transducers, while the width was chosen to create an area large enough for safe illumination of the skin.

#### 2.2.1 Laser

Functional optoacoustic imaging of oxygenated and de-oxygenated hemoglobin was achieved using a dual wavelength laser system made in house by Seno Medical Instruments and comprised of Nd:YAG laser operating at the “long” NIR wavelength of 1064 nm and emitting 15 ns long pulses with the pulse energy of 150 mJ, and Alexandrite laser operating at the “short” NIR wavelength of 757 nm and emitting 50 ns pulses with pulse energy of 140 mJ [35]. Functional imaging at two wavelengths co-registered in time requires that the delay between pulses emitted at the short and long wavelengths is reduced to about 5 ms to minimize the effect of tissue motion. The cycling pairs of laser pulses are delivered with the repetition rate of 5 Hz.

The design of this laser system incorporated a common, customized power supply and electronics to facilitate rapid, alternate pulsing of the two lasers while synchronizing the optical pulsing with the ultrasound system within the Imagio™. Some of the technical challenges included maintaining a reasonable size and cost for a commercially viable system, creating a light path combiner, and minimizing electrical noise for the required high signal -to-noise ratio of the ultrasound detection system.

#### 2.2.2 Probe protection and light delivery design

The LASER light is delivered to the probe *via* a bundle of 200 μm diameter optical glass fibers, and then spread uniformly across the two 40 mm × 6 mm output windows using custom lenses and optical diffusers. While a significant number of thin optical fibers is required to achieve the desired flexibility and durability of the bundle, it is important to have these fibers to be fully randomized on the output, so that no two neighboring fibers on the input are placed in proximity of each other on the output. We found 8.3 mm an optimum input diameter of the fiber bundle for fully randomized and uniform output that is safe for the patients. The peak laser intensity at the input of the fiber bundle was <5 MW/cm^2^ for the Alexandrite laser and 35 MW/cm^2^ for the Nd:YAG laser, which is significantly lower than the damage threshold level of ∼0.45 GW/cm^2^ measured for our glass fiber bundles with hot-fused input tip.

Our design of the fiberoptic light delivery system aimed (i) to maximize the optical energy that is delivered to the tissue and (ii) to ensure that light passing through the components of the probe is not absorbed by those components as any absorption there would generate an OA signal in close proximity to the probe’s transducers which, in turn, would create a large, unwanted signal response. The of light incidence angle influence on the optoacoustic contrast of lymph nodes was studied in [39], and the authors concluded that the optimal angle of maximum contrast depends on the depth and dimensions of the objects. Our own conclusions were in agreement with [39] and other works that studied the design of hand-held optoacoustic probes. The light delivery angle of incidence to skin surface as well as the distance between the optical windows and the stack of ultrasonic transducers in the probe of Imagio™ was optimized for tumors with dimensions of about ∼10 mm and the depth of about ∼20 mm. Nevertheless, tumors with dimensions of 3-to-20 mm as well as small and large blood vessels could be visualized by Imagio in the range of depths from right under the skin to 40 mm.

The materials and components of the probe were selected and designed to minimize internal signals generated inside the probe housing. Additionally, a portion of the transmitted light is reflected from the skin and would also create an unwanted signal response from the acoustic lens. This effect was minimized by doping the transducer acoustic lens with a white reflective powder of TiO_2_ and then applying a thin gold film to the exterior of the acoustic lens, both of which reflect back practically all light incident upon the lens. The lens materials must also assure maximum transmission of the desired acoustic signal to the ultrasonic transducers and were selected and designed accordingly.

**Fig. 3:**
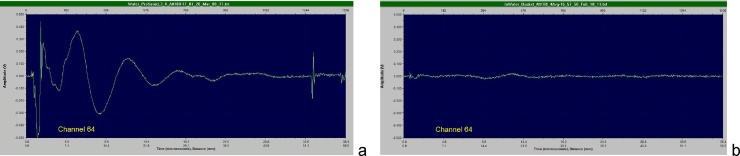
Optoacoustic signals recorded with a hand-held optoacoustic probe with (a) no protective features and with (b) protection of the acoustic lens from the incident optical pulses and with protection of the ultrasonic transducers and probe housing from the light leaked out of the optical fibers.

**Fig. 4:**
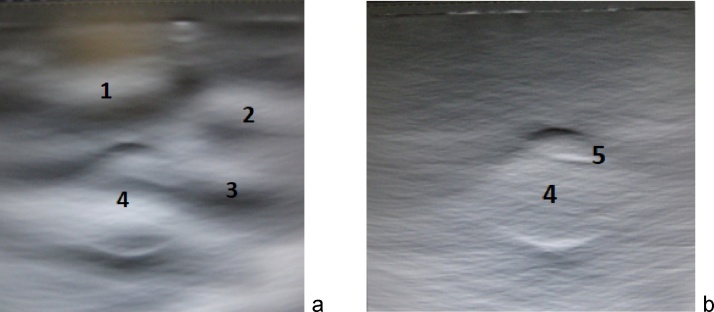
Optoacoustic image of a tissue phantom acquired using: (a) an unprotected hand-held probe and (b) a “protected” optoacoustic image of the same phantom acquired with a probe protected from laser generated acoustic artefacts and OA signals filtered with a high-pass filter >100 kHz.

Fig. 3 shows an optoacoustic signal detected by the optoacoustic probe with a number of high frequency and low frequency artefacts prior to the advances described above and a clear signal obtained after the advanced design of the probe was implemented. Fig. 4 depicts two optoacoustic images of a breast tissue mimicking phantom made of polyvinyl-chloride-plastisol (PVCP) [40,41] with a spherical inclusion having relatively low optical absorption of μ_a_∼0.2/cm. When no bright objects (such as blood vessels) are present in the image plane, optoacoustic artefacts can be prominently visible (Fig. 4a). However, protection of the acoustic lens from the laser light diffusely scattered by skin towards the probe and simultaneous protection of the ultrasonic transducer stack and the probe housing from the light leaking from the fiberoptic light delivery system along with a high-pass filer removes the laser-generated artefacts (Fig. 4b).

**Fig. 5:**
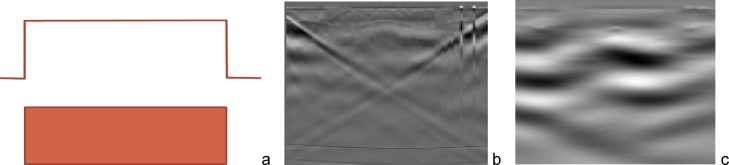
(a) Sharp rectangular profile of the optical beam from an optoacoustic hand-held probe causes V-shaped artefact on the sonogram of optoacoustic signals, and (b) low frequency wavy artefact objects on corresponding optoacoustic images.

When illuminating skin through windows in the optoacoustic probe, it is natural to provide the most homogeneous beams of light with well-defined sharp edges. Sharp edges of the beam of optical energy can cause very strong optoacoustic artefacts. As shown in Fig. 5a–c sharp edges the optical beam abruptly ending in proximity of transducer element 1 and element 128 of the 128-element linear ultrasonic transducer array generate sharp acoustic pulses propagating laterally through the skin from edges to the center of the probe and being detected sequentially by the element numbers 1,2…64 and 128,127…65. This sequential detection appears as a V-shaped artefact signal on the optoacoustic sinogram (Fig. 5a) and as a low frequency waves” on the corresponding optoacoustic image (Fig. 5b). Optical illumination with beams having smooth Gaussian edges alleviates these V-shaped artefacts on the optoacoustic images.

Although the probe is designed to maximize the reflection of incident light, it must minimize the reflection of incident ultrasound. If any ultrasound pulses are reflected back into the tissue they will create additional reflections (artefacts) from the tissue back to the transducer.

These reflections, or reverberations, will create additional acoustic noise reducing OA image contrast. The transducer acoustic design and receiver are optimized to have a minimum acoustic reflection coefficient through careful selection of lens material, matching layers, backing material and receiver impedance. The hand-held probe containing linear array of ultrasonic transducers was designed to provide sensitivity for both low and high ultrasonic frequencies that enable high volumetric contrast and high spatial resolution, while also minimizing optoacoustic artefacts. These advances assured satisfactory clinical performance of Imagio™ system in multicenter clinical trials [8].

#### 2.2.3 Image contrast *vs* resolution

When breast vasculature and dense microvasculature of malignant tumors absorb a short pulse of optical energy, the central frequency of the optoacoustic response is inversely proportional to the size of the tissue structure and, since there are widely varying sizes of vasculature, the bandwidth of the ultrasonic transducers must accommodate the smallest to the largest anticipated sizes. The sensitivity bandwidth of our system was from 0.1 MHz to 12 MHz at -6 dB level, which is termed ultra-wide band [12]. Such bandwidth was achieved by utilization of a proprietary single crystal piezoelectric composite material of the ultrasonic transducers, accurate optimization of the acoustic impedance of the backing material and the design of the front matching layers, as well as the electrical coupling of the ultrasonic transducer output to the high-impedance (>50 kΩ) input of the preamplifier. Excellent sensitivity of the piezoelectric composite combined with ultralow noise electronic microcomponents provided the noise equivalent pressure of the optoacoustic signal amplitude detection, NEP∼1.3 Pa.

**Fig. 6:**
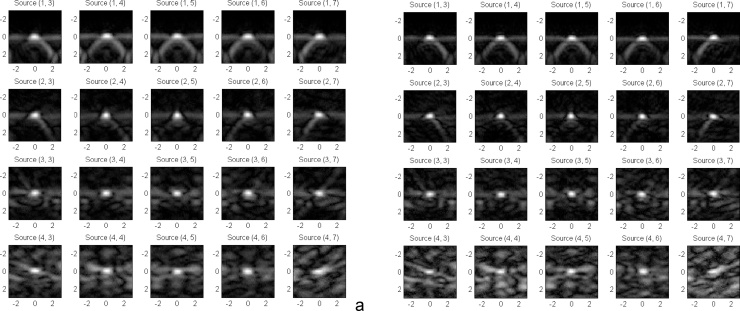
Demonstration of spatial resolution as a function of depth for two laser pulse durations: (a) Alexandrite laser with 50 ns long pulses and (b) Nd:YAG laser with 15 ns long pulses. All images were acquired in after a single laser pulse illumination. Source (k,n) means thread number in the grid matrix of k = 4 and n = 7.

The axial (depth) resolution, Fig. 6a shows optoacoustic images of the threads in the phantom grid acquired with Alexandrite laser (pulse duration ∼50 ns, wavelength 757 nm). Fig. 6b shows optoacoustic images of the threads in the phantom grid acquired with Nd:YAG laser (pulse duration ∼15 ns, wavelength 1064 nm). The axial resolution was found to be 0.47 mm for Alexandrite laser and 0.42 mm for Nd:YAG laser. The lateral resolution was found to be the highest in the center of the field of view for Imagio™ and equal to 0.81 mm for Alexandrite laser and 0.73 for Nd:YAG laser. The lateral resolution degrades with decreasing acoustic aperture of the probe if the object is located in the far zone [42]. The lateral resolution of an optoacoustic system also depends on directivity of the individual ultrasonic transducers, which limits the acoustic aperture of the array and, consequently, its lateral resolution if the object is located in the close proximity of the transducer array [43]. Therefore, as shown in Fig. 6, the lateral resolution of the OA image in close proximity of the probe (near zone) and at a depth equal or greater than 3 cm (far zone) was decreased to ∼1 mm for Alexandrite laser and ∼0.92 mm for Nd:YAG laser. The image contrast at the depths greater than 3 cm was simultaneously reduced.

### 2.3 Signal processing

The initial signal processing sensitivity is used to compensate for variability in the intensity of the signals measured from each channel. A zeroing of the bad elements (if any), which were detected earlier, is also performed. One main purpose of signal processing in an optoacoustic imaging system is to restore the intrinsic profiles generated in tissues by short laser pulses. The optoacoustic signals detected by a system operating in the backward mode (such a the hand-held probe based Imagio) were analyzed by Karabutov et al [44]. The signals detected by acoustic transducers in the array consist of the two components: (1) The optoacoustic signal represented by a N-shaped pulse generated by an absorbing target (such as a blood vessel or a microvascular cluster) superimposed on the slope of the effective optical attenuation in the breast, (2) white thermoelectrical noise exhibited as high frequency fluctuations. The major source the OA signal distortion is the imperfection of the sensitivity spectrum of the optoacoustic transducers. The detected optoacoustic signal $p\left(r_{T},t\right)$ generated by a transducer, T, with an active area $S_{R}(r_{T})$ centered at the transducer position, $r_{T}$, can be expressed as [45]:

(1) $$p\left(r_{T},t\right)=h^{eir}\left(t\right)*\frac{1}{S_{R}\left(r_{T}\right)}\int_{S_{R}\left(r_{T}\right)}^{}{dS}_{R}\left({r^{'}}_{T}\right)\;p\left({r^{'}}_{T},t\right),$$

where $h^{eir}\left(t\right)$ is the acousto-electric impulseresponse (EIR, V/Pa), and denotes a 1D temporal convolution. We employed Wiener deconvolution of EIR from detected optoacoustic signal to restore their intrinsic spectrum of ultrasonic frequencies [46].

The ultrasonic transducers in the probe have finite dimensions, and therefore, the optoacoustic signals detected at oblique angles of incidence are distorted by the pressure averaging effect over the transducer’s active area. The optoacoustic signal produced by an ultrasonic transducer centered at location **r**_T_ that arises from a thermoacoustic point emitter at location **r** can be expressed as:

(2) $$p\left(r_{T},t\right)=h^{eir}\left(t\right)*h^{sir}\left(t\right)*\left(\frac{\beta }{2\pi C_{p}}\frac{d}{dt}\frac{\delta (t-\left|r_{T}-r\right|/c_{S}}{\left|r_{T}-r\right|}\right),$$

where the last term in the round brackets describes an optoacoustic signal detected by an ideal point-like transducer from a hemisphere of optoacoustic pressure emitting voxels, and $h^{sir}\left(t\right)$ is the spatial impulse response that describes the sensitivity spectrum of the optoacoustic transducers as function of the angle of optoacoustic wave incidence.

Each of the 128 transducer elements within the array had width of 0.25 mm, which was sufficiently narrow to enable wide directivity within the image plane in the frequency range relevant for both, optoacoustic and ultrasonic tomography. Deconvolution of the spatial impulse response (SIR) from the detected optoacoustic images further contributed to the widening of the angular directivity of the transducer array and alleviated the effect of the finite width of the transducers on the lateral resolution.

The thermoelectrical noise spectrum is typically outside the spectrum of ultrasonic frequencies present in the optoacoustic signals from breast tissue structures. Therefore, a bandpass filter with its max frequency cutoff selected just outside of the low frequency cutoff of the thermoelectric noise, and its low frequency cutoff selected at about 67 kHz permitted high contrast detection of small and even larger (∼15 mm) tumors.

### 2.4 Image reconstruction

Based on the optoacoustic signals received at each transducer element, the distribution of the absorbed optical energy in the illuminated volume is reconstructed using a modified filtered back-projection algorithm for 2D tomography. The essence of such algorithm is the integration of the optoacoustic signals over the voxels on the spherical surfaces, r, within tissue contributing to each sample of the optoacoustic signals at a given time, $t=r/c_{s}$. In early works, a number of closed form analytical filtered back-projection formulas have been developed for spherically expanding optoacoustic waves by adopting inversion of Radon transform originally employed for computed (x-ray) tomography based on planar waves [[47], [48], [49]]. The analytical methods utilizing filtered back-projection are practical for real-time imaging, especially in clinical applications. However, these algorithms are approximate since in the tomography systems based on hand-held probes with limited acoustic aperture, there are incomplete data sets. On the other hand, with homogeneous distribution of the optical illumination through the field of view of the probe, relative brightness of tissue structures located in various parts of the image can be compared, and the accuracy is proven to be sufficient for the real-world practical clinical application in diagnostic imaging of breast cancer.

A reconstruction algorithm performs a back-projection of filtered and weighted OA signals taking into account the transducer EIR (frequency response) and SIR (angular response). When an incomplete set of optoacoustic signal data is acquired through a portion of the 2π solid angle, general analytic reconstructions, such as the filtered back-projection algorithm offers an approximate reconstruction of the original pressure distribution, $p_{0}\left(\vec{r}\right)$ of the optically induced acoustic sources [50]

(3) p0r→=Γr→μar→F(r→)=2Ω0∫sdΩpr→',t-t∂pr'→,t∂tt=r→-r'→/c0

where $\Omega_{0}<2\pi$ is the solid angle of the entire detection surface, S with respect to a given source point located at r, $p\left(\vec{r},t\right)$ is the pressure received at detecting position r and time t. Eq. (3) indicates that $p_{0}\left(\vec{r}\right)$ can be obtained by back-projecting the filtered data function $\left[p\left(\vec{r},t\right)-t\frac{\partial p\left(\vec{r},t\right)}{\partial t}\right]$ onto concentric spherical surfaces centered at each ultrasonic transducer, $r_{T}$. The magnitude of each data sample is then normalized to the factor, $Rd\Omega /\Omega_{0}$, which applies proper weight to voxels located at a distance, R, from the ultrasonic transducers and visible within element aperture  dΩ of each transducer relative to the total aperture of the array, $\Omega_{0}$. Contribution of the first derivative of the pressure signal, $p\left(\vec{r},t\right)$, over time, $\frac{\partial p\left(\vec{r},t\right)}{\partial t}$ is similar to the ramp filter in the frequency domain, which provides shaper edges of the larger objects. The use of the volumetric 3D Eq. (3) for filtered back-projection reconstruction of 2D images is heuristic, justified by the facts that (i) the optoacoustic signals are detected from the laser illuminated volume under the hand-held probe and not just the image plane (as low ultrasonic frequencies that carry the main energy of the optoacoustic brightness of tumors cannot be rejected by the acoustic lens) and (ii) in our signal processing we account (among other factors) for the spatial impulse response of each transducer in the array.

### 2.5 Image processing

Back-projected optoacoustic images without image processing compensation would otherwise display gradually decreasing brightness of voxels as a function of depth. To quantify this attenuation, we determined bulk breast tissue optical absorption coefficient $\mu_{a}^{b}$ [cm^−1^], through analyzes of references [[51], [52], [53], [54], [55], [56]] and experimentally measured optoacoustic profiles representing the optical fluence attenuation in patients. The average numbers were as follows: 0.039 ± 0.011 at 757 nm and 0.122 ± 0.016 at 1064 nm. The effective optical scattering coefficient, ${\mu '}_{s}^{b}≃{\mu '}_{s}^{T}$ for both tumors and normal breast tissue was found to be ∼7.0-11.0 cm^−1^ at 757 nm and ∼3.5 – 4.5 cm^−1^ for 1064 nm. Optical absorption coefficient of cancerous breast tumors $\mu_{a}^{T}$ [cm^−1^] was found to be statistically 2- to 3-fold higher (0.130 ± 0.060 at 757 nm) than in the surrounding breast tissue, but only, only slightly higher (0.154 ± 0.089) at 1064 nm. The average tissue optical properties result in an average effective attenuation of the optical flux in the near-infrared spectral range of approximately 3 times per cm.

Acoustic attenuation has been studied by Foster et al [57]. Based on this study and the breast composition reported by Geise et al. [58] we defined an average acoustic attenuation of the breast. Using a 70/30 ratio between fatty and fibroglandular tissue, we obtained the following formula:

(4) $$u\left(z\right)=u_{0}10^{-\alpha_{us}f_{oa}^{1.5}z}$$

where $\alpha_{us}=0.32$ [dBcm MHz] is the coefficient of ultrasound attenuation in the breast. Based on eq. (4), ultrasound at 10 MHz is attenuated by the breast ∼3 times, as strong as the pulse of near-infrared light, which was consistent with our analysis of the initial clinical data.

Analysis of the optical and acoustic properties of the breast shows that breast tissue effectively attenuates both near-infrared light and ultrasound. Therefore, the first step in the optoacoustic image processing was accounting for OA signal attenuation.

We developed a method of the OA image brightness normalization that resulted in the equal brightness of the well-characterized objects on clinical images of the breast, such as artery cross-sections. The method includes the following steps: (i) segment the brightest objects on the image: blood vessels and tumors, (ii) calculate average pixel brightness in each horizontal row of pixels excluding that of segmented objects, (iii) normalize each pixel brightness by dividing it to the average value at each depth.

**Fig. 7:**
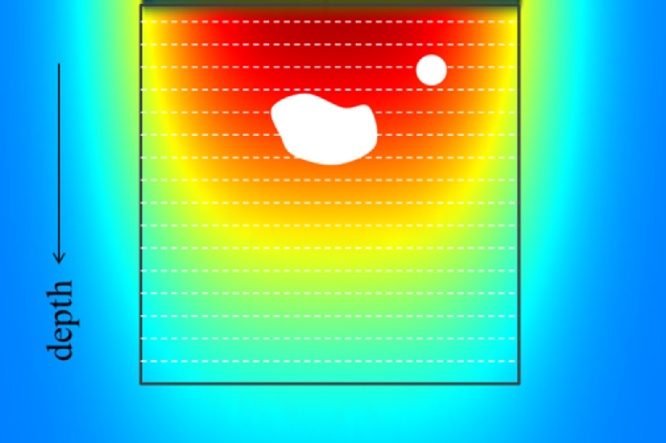
Image depicting the method of background removal through segmentation, calculation of average background brightness and row by row of pixel brightness normalization.

To test this method, it was applied to an optical and acoustically simulated dataset. Fig. 7 shows an image of Monte Carlo simulated light propagation and attenuation as a function of depth where artery and a tumor segmented and removed from the image. Average optical properties of the breast [[52], [53], [54], [55], [56]] and blood [28] were used to perform this routine computer simulation. Horizontal dashed lines represent horizontal rows of pixels average brightness of which was calculated and used as a normalizing divider parameter for this row of pixels. The resulting 2D image is depth independent.

### 2.6 Image display

**Fig. 8:**
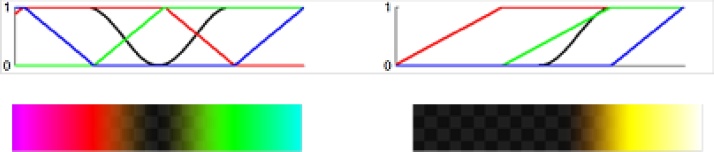
Design of the color palette for display of functional OA maps of [sO2] (left) and [tHb] (right) calculated relative to the average background value after segmentation and removal of bright objects such as blood vessels and tumors.

Imagio produces images for each laser wavelength (757 nm, known as OA Short and 1064 nm known as OA Long). First a reference region is selected. Then the two optoacoustic images are converted into the three functional maps: one map of the total hemoglobin, [tHb], proportional to the density of microvasculature in breast tissues, and 2 different maps of blood oxygen saturation, [sO2] relative to its average background level. The color palette for display of functional images is designed to reveal the functional information to the radiologist in its easy to interpret color map. The RGBA color maps are used for the [sO2] (left) and [tHb] (right) as displayed in Fig. 8 below. RGB colors are used to display only maximum image brightness above an empirically defined threshold. The image transparency values are between 0 and 1. To compute the color, the intensity of a pixel from the estimated map data is offset by the reference level and scaled in proportion to the standard deviation of the reference region, and the result is mapped onto a color value. This is called statistical mapping [59]. The zero opacity channel (black curve in Fig. 8) is used to achieve pixel transparency for the lower [tHb] values and median [sO2] values that are sub-threshold. The transparency occurs when values of the optoacoustic functional images are below a defined level. The role of transparent OA pixels is to provide a vivid display of gray scale values of B-mode ultrasound co-registered with OA image pixels.

As depicted in Fig. 8 (left), functional maps of [sO2] are presented in a red/green palette. The red color indicates blood oxygen saturation below the typical [sO2] level in normally oxygenated tissues (85%) and goes down to typical blood oxygenation in veins and even lower. The extreme [sO2] values of hypoxic blood (lower than typical venous blood) are indicated with magenta color, which is a characteristic of an extremely hypoxic tumor. For a clinical interpretation of an image, red color within tumors means that levels of relative deoxygenation is present, which is one of the indications for suspicion of malignancy”. The green color, on the other hand, indicates blood oxygen saturation typical of normally oxygenated tissues (>90%). The extreme [sO2] values of arterial blood is indicated with green turning into cyan. As depicted in Fig. 8 (right), functional maps of the total hemoglobin [tHb] are presented in yellow palette. Only the highest relative values of [tHb] are colored, such as high density of microvasculature or larger individual blood vessels. The threshold is selected to make all tissues with normal concentration of microvasculature transparent for clear display of tissue anatomy on background ultrasound images. To summarize, the colored pixels on functional [sO2] images are presented in certain areas when these areas are much brighter on one of the optoacoustic images (acquired at 757 nm or 1064 nm), and the pixels of functional [tHb] images are colored when there is strongly bright area on at least one, but often both (757 nm and 1064 nm) optoacoustic images.

In addition to demonstrating relative degrees of oxygenation-deoxygenation, OA can demonstrate normal and abnormal anatomy of vessels that lie within a breast mass and its surrounding tissues. Interpreting physicians can assess the anatomy of these OA-demonstrated vessels in addition to the degree of relative oxygenation-deoxygenation in order to refine their overall evaluation for risk of malignancy.

### 2.7 Co-registration of optoacoustic and ultrasonic images in real time

The co-registered functional color OA maps are interleaved temporally and presented simultaneously in real-time along with the gray scale (B-mode) ultrasound. Co-registration of optoacoustic and ultrasound images is performed with the video frame rate of 10 OA/US images per second (5 fps for each of the two laser wavelengths). Since the creation of a single complete OA frame requires a pulse from each of the 2 OA wavelengths, complete OA frame rates are 5 fps rather than 10 fps. The two lasers (see section 2.2.1) operating independently were synchronized to emit a pair of pulses at the wavelengths of 757 nm and 1064 nm with a short delay of 5 ms between them. During such a short period of time almost no motion occurs in the course of hand-held probe scanning on the skin surface of the breast, even when the probe is moved slowly across the breast. Thus, when the duration separating data acquired from each wavelength is small, the multi-wavelength pixelwise operations for conversion of optoacoustic images into functional images do not produce motion artefact noise. There is a difference in frame update rates of gray scale ultrasound and OA. The ultrasound frame rate varies with depth of field and other technical settings, but is generally at least 3 to 4 times the update rate of the OA images. While the OA images are robust enough to tolerate slow sweeping of the transducer across the breast during scanning, movement of the transducer that is too fast can result in slight temporal and spatial miss-registration of the OA images with the underlying gray scale ultrasound images. Though a potential problem, in clinical trials, this was dealt with merely by adequate training in scan techniques, and was found not a clinically significant problem [8].

The computer display shows separately an anatomical ultrasound image and two functional images of [sO2] and [tHb] superimposed with the anatomical image. All images are presented on screen simultaneously, but in clinical studies, the readers evaluated and interpreted the images in the following sequence: (1) B-mode ultrasound, a conventional image of breast morphology that gives the first diagnostic indications to the radiologists, then (2) functional image of [tHb] superimposed with ultrasound, and (3) functional image of [sO2] superimposed with ultrasound. The commercial version of the developed imaging system displays the two optoacoustic images (relative [sO2] and total [tHb]), plus a “combined” image of [sO2] where only those pixels are colored that have colors on the [tHb] image. Thus, the total number of images on the display panel is six, when conventional ultrasound, OA Short (757 nm) and OA Long (1064 nm) are also included. However, for the purposes of Imagio technology presentation here we discuss only the main 3 images on the display that play the key role in the diagnostic imaging of breast cancer. In summary, the co-registering and temporal interleaving of OA and gray scale ultrasound images is creating a real time oxygenation-deoxygenation blood map fused with an underlying gray scale anatomic image.

## 3 System validation

### 3.1 Imaging of phantoms with well-defined properties

**Fig. 9:**
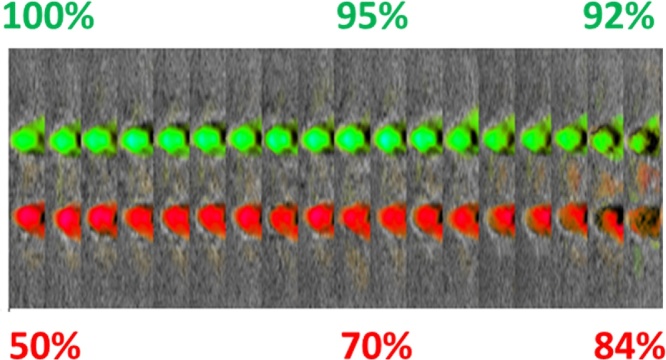
Functional images of oxygen saturation in the flowing blood with gradually changing percentage [sO2] values. Images were acquired from two 2.4 mm diameter tubes placed in a breast mimicking PVCP phantom with matching optical and acoustic properties. The colors depicted match the design of the color palette of Fig.8. All images were acquired with a single laser pulse illumination.

The capability of Imagio™ to display functional values of [sO2] relative to the median background values of normal breast tissues was studied in a phantom made of PVCP with optical and acoustic properties replicating average breast tissue [40,41] and containing two tubes with diameter of 2.4 mm, placed at 10 mm depth and filled with continuously flowing bovine blood having hematocrit of 27%. Optoacoustic images of this phantom were acquired with video frame rate at the 2 laser wavelengths of 757 nm and 1064 nm, and [sO2] images of the two blood vessels were reconstructed and shown below in Fig. 9 superimposed on the ultrasound image background. Fig. 9 represents a series of images (two tubes each) which are placed orthogonal to the probe and assembled adjacent to each other. The purpose is to better observe the transition of relative colorization in a single image. Thus, all tubes shown in the image are at the same depth of 10 mm.

Blood oxygenation was changed by gradual oxygenation of fully deoxygenated blood in the air. The level of [sO2] in the tubes was monitored with a commercial oximeter. In this experiment, blood in the top vessel was changed from ∼100% [sO2] level to ∼91% level, while [sO2] in the other vessel was varied from ∼50% to 85%. Fig. 9 displays 18 images of the pair of tubes as [sO2] levels were varied and it demonstrates expected functional colors. Even though OA/US clinical system is designed to display qualitatively only ranges of [sO2] values relative to the median background level of normal tissues, our experiments aimed at validating accuracy of relative blood oxygen saturation in tissue phantoms with real flowing blood. We observed [sO2] colors according to the design of the color palette (see Fig. 8), *i.e.* green color images were observed in the [sO2] range of ∼100% to ∼91%, no color is the [sO2] range from ∼90% to ∼85%, and red color images are in the [sO2] range below ∼85%.

**Fig. 10:**
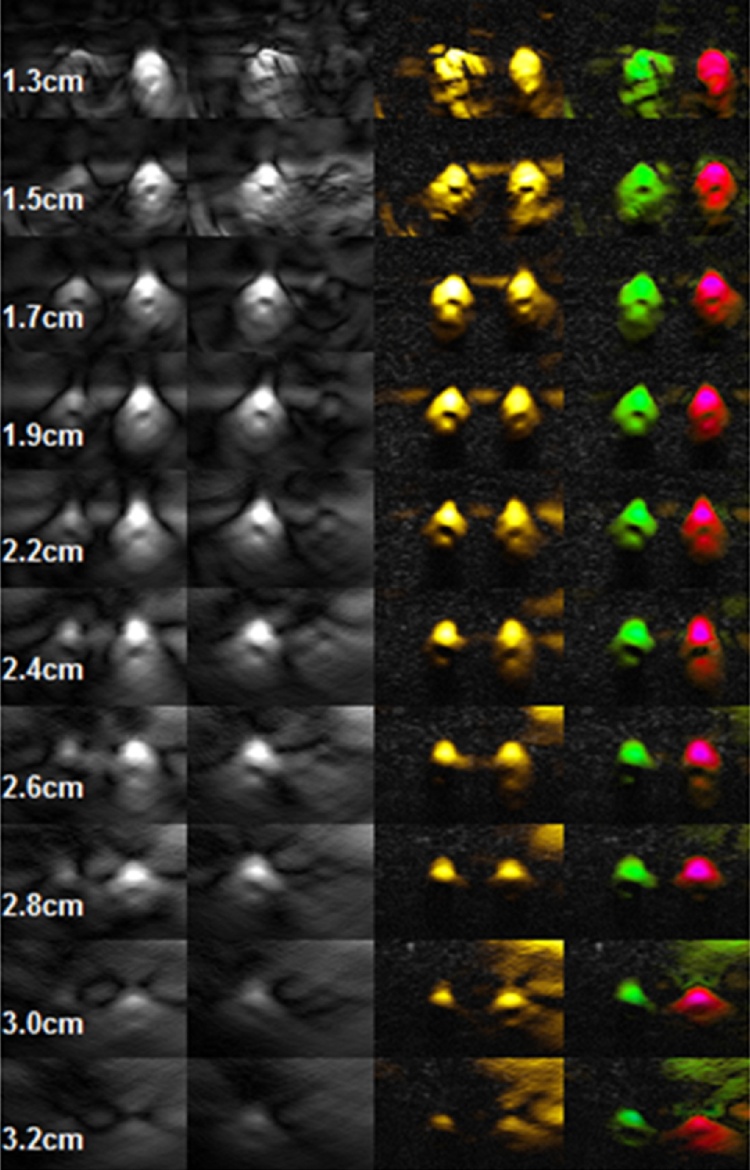
Optoacoustic and functional images of two 1.5 mm diameter tubes with flowing blood positioned in an aqueous phantom with optical properties of an average breast. Columns from left to right depict the following images: OA image acquired at 757 nm, OA image acquired at 1064 nm, functional image of [tHb], functional image of [sO2]. All images were acquired with single laser pulses.

Fig. 10 demonstrates the system capability to correctly display [sO2] colors with gradually increasing depth of blood vessels. Two tubes with flowing diluted bovine blood having hematocrit of 27% and fixed level of oxygen saturation (50% in the right tube and 100% in the left tube) were placed in an aqueous phantom (a tank with milky water) simulating optical properties of the breast. The depth of tubes was varied from 13 mm to 32 mm. Typically, the maximum depth of breast tumors is limited to about 3 cm for a patient who is scanned while lying in the supine or posterior oblique position.

This phantom experiment demonstrated that brightness of the optoacoustic images and colors of functional images are displayed correctly through the entire range of depths in the phantom. A gradual decrease of the image contrast was observed with increasing depth of the tubes from the imaging surface.

**Fig. 11:**
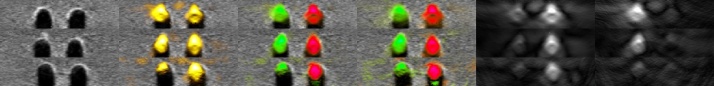
Cross-sectional mages acquired by OA/US system from two 2.4 mm diameter tubes with flowing blood in a breast mimicking PVCP phantom. From left to right (6 columns) ultrasound images. From top to bottom: tubed with blood having hematocrit of 41% (top row), 27% (middle row), 4% (bottom row).

Fig. 11 shows the system capability to display functional colors for various levels of blood concentration (hematocrit, Ht) from 41% (full blood) to 27% (diluted blood) to 4% (strongly diluted blood). A breast mimicking PVCP phantom was created with two horizontal tubes positioned at the depth of 20 mm.

The results obtained in the phantom were used for validation pertaining to the system readiness for clinical studies. Not only did the system accurately present colors representing the blood oxygen saturation in the major vessels, the image [sO2] color did not switch or disappear in the case of low hematocrit, however [tHb] signal-to-noise ratio became weaker against the background at the lowest hematocrit levels. In the clinical study of tumor angiogenesis, it has been found that 70-μm to 150-μm size vasculature can show significant deoxygenation in aggressive solid tumors [22,23]. This size is below the resolution of the hand-held OA probe and the average density of tumor microvasculature can make tumor contrast 5–10 times lower, compared with blood vessel. Nevertheless, given the high contrast between hemoglobin and surrounding tissue, and the high contrast between the alternating laser wavelengths, the OA system is able to resolve this oxygenation difference even with the average concentration of blood 10-fold lower than that inside of blood vessels.

### 3.2 Clinical studies in patients

During the device investigation, several different clinical studies were conducted on patients. Following the system validation in phantoms, a clinical feasibility study was performed on patients with breast masses suspected for cancer based on screening mammography followed by breast ultrasound. Patients with Breast Imaging Reporting and Data System (BI-RADS) scores of 3, 4 or 5 were recommended for dual-modality functional anatomical imaging comparing noninvasive diagnosis from the OA and US co-registered images of Imagio™ with the gold standard of core biopsy. Based on the clinical feasibility data of an initial 79 patients, the system was calibrated and its functional imaging algorithm was trained, which means that the thresholds were determined for display of colors in the area of image maximum brightness of the total hemoglobin [tHb] distribution and its oxygen saturation [sO2]. Following the feasibility study, a multicenter clinical trial guided by the Food and Drug Administration was performed on over 2000 patients to determine sensitivity and specificity of noninvasive diagnosis of breast masses suspected for malignancy utilizing the co-registered optoacoustic functional and ultrasound morphological images. The statistical analysis of this clinical trial was reported in [8]. Here we report technological features and advances of the OA/US imaging system that enabled clinical viability of Imagio™. The clinical examples described below were selected to demonstrate how the functional - anatomical imaging of optoacoustic imaging of Imagio™ improved the specificity of breast cancer detection by radiologists compared to traditional conventional ultrasound.

**Fig. 12:**
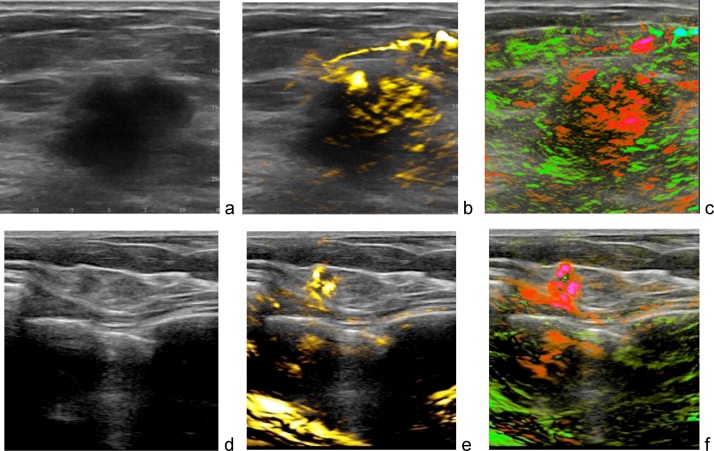
Two examples of OA/US images of breast carcinoma in two different patients. Upper row shows a 2.6 cm malignant mass on gray scale ultrasound (a) with increased internal total hemoglobin due to high density of angiogenesis in more than half of the tumor (b) and diffuse internal blood deoxygenation (c). The bottom row shows a small 4 mm grade II invasive ductal carcinoma with relatively low contrast on gray scale ultrasound (d), however with high density of angiogenesis around a small core on OA total hemoglobin map (e) and significant blood hypoxia on [sO2] map. Note that there is dense angiogenesis microvasculature with significant deoxygenation (red) within these masses and their margins. Also, the upper row Fig.12c shows numerous green radiating parasitized feeding arteries in the tissues that surround the mass – additional image feature indicating pathology of malignancy. Images are 38 × 38 mm.

**Fig. 13:**
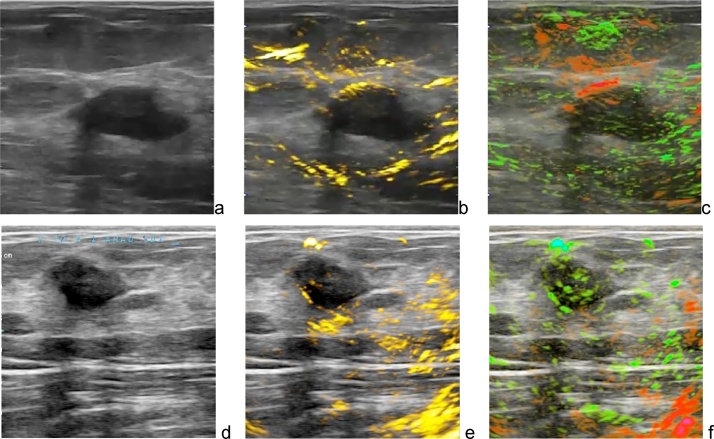
Two examples of OA/US images of benign fibroadenoma. Optoacoustic functional images invert the conclusion based on ultrasonic morphological image, downgrading BIRADS to 3. Images are 38 × 38 mm.

**Fig. 14:**
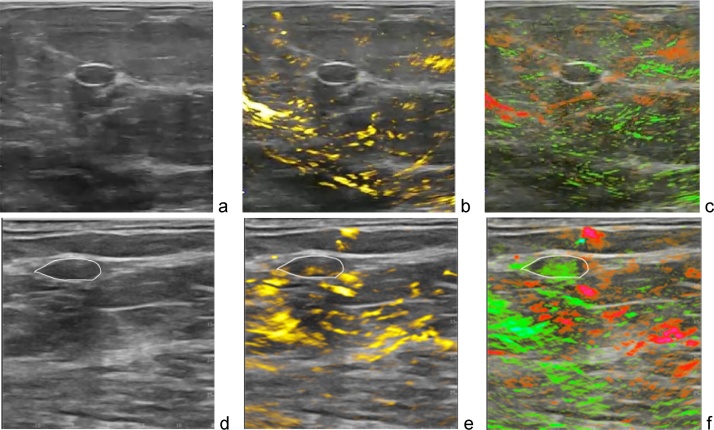
Two examples of OA/US images of benign fibroadenoma. Information from optoacoustic functional images reaffirm radiologist interpretation of the mass being probably benign. Images are 38 × 38 mm.

**Fig. 15:**
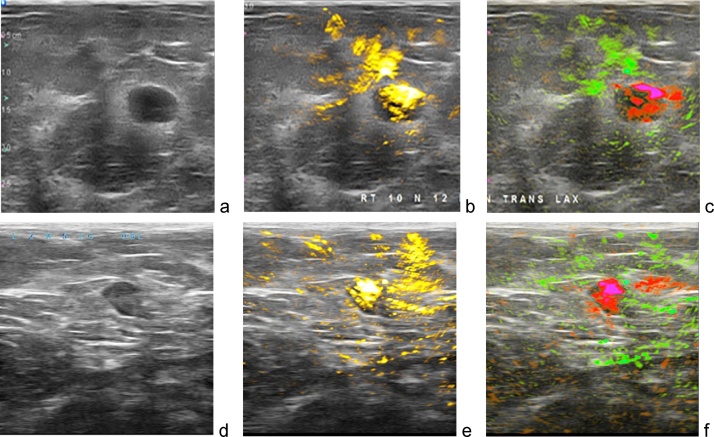
Two examples of OA/US images changing the diagnosis from probably benign solid masses to masses suspicious for carcinoma. Optoacoustic functional images upgraded radiologist interpretation to BI-RADS 5 (highly suspicious for malignancy) from BI-RADS 3 (probably benign) based on conventional ultrasonic morphological images. Biopsy confirmed malignancy in both patients. Images are 38 × 38 mm.

**Fig. 16:**
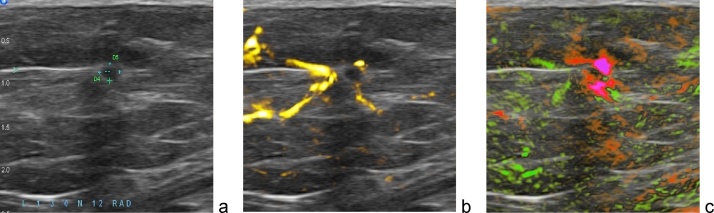
An example of OA/US images of breast carcinoma *in situ*. Optoacoustic functional images demonstrate radially oriented vessels extending in to the peripheral zone, upgrading BIRADS 3 to BIRADS 5. Biopsy confirmed cancer. Images are 25 × 25 cm.

Fig. 12, Fig. 13, Fig. 14, Fig. 15, Fig. 16 present a series of OA/US images obtained from patients with malignant and benign masses. These images were all acquired prior to the core needle biopsy, which defined the final pathological outcome. The patients enrolled in the clinical study were present with masses determined to be suspicious for cancer based on finding discovered by mammography, clinical exam, or imaging findings other than from gray scale ultrasound. Mammogram sensitivity of detection of breast cancer is reduced in patients with dense and heterogeneous breast tissue [60,61]. Breast ultrasound exam is often performed as a complementary study to diagnostic mammography. The diagnostic capability of breast ultrasound is based on gray scale display of tissue morphology. The main factors that influences BI-RADS classification of a mass on ultrasound imaging are its morphologic features of shape, echotexture, sound transmission, and margins. Irregularly shaped or masses, those with heterogeneous internal texture or internal microcalcifications, acoustic shadowing, or indistinct or frankly speculated margins occur with malignant masses, while round or well-circumscribed shape more often indicate benign mass. However, gray scale features of benign and malignant features overlap greatly, and interpreting physician have to err to the side of caution, favoring suspicious diagnoses and accounting for the low specificity of gray scale ultrasound. Panels a and d of Fig. 10, Fig. 11, Fig. 12, Fig. 13, Fig. 14 show diagnostic B-mode ultrasound anatomical images of selected breast masses. The method of cancer differentiation from benign tumors based on functional images of [tHb] is based on the premise that aggressively growing malignant tumors possess dense areas of angiogenesis-related microvasculature. Additional diagnostic factors based on vasculature include absence of active angiogenesis and vasculature that radiate toward and away from tumors (no penetration, recruitment or drainage). Panels b and e on Fig. 10, Fig. 11, Fig. 12, Fig. 13, Fig. 14 show in yellow total hemoglobin map the areas of the highest density microvasculature and the major vasculature. The method of cancer differentiation from benign tumors based on functional images of [sO2] is based on the premise that aggressively growing areas of malignant tumors are hypoxic. Rapid proliferation of cancer cells and faster metabolic rate of oxygen consumption in aggressive cancer results in reduced blood oxygen saturation measured in the tumor microvasculature [62]. In addition, using colors it is easier for the radiologist to visualize feeding arteries and draining veins in the tumors and surrounding areas. Panels c and f on Fig. 12, Fig. 13, Fig. 14, Fig. 15, Fig. 16 show in green/red relative oxygenation-deoxygenation map the areas of tissues with normal blood oxygen saturation (above 90%) and hypoxic areas (below average [sO2]∼85% level of normal background tissue). The images of blood oxygen saturation also clearly differentiate green-color vessels and red-color vessels. The simultaneous qualitative functional imaging assessment of the tumor vascular and microvascular environment provides much more confidence to the reading radiologist when the functional images are overlaid with breast morphology. Without the anatomical morphological background of conventional ultrasound, there would be much less confidence in the colored functional parametric maps.

Fig. 12 gives two examples of how optoacoustic functional images enhance the confidence of radiologist interpretation of breast carcinoma based on ultrasound. Fig. 12a depicts an irregular intensely hypo echoic solid mass with indistinct margins. This is suspicious for malignancy by gray scale ultrasound. Fig. 12b shows a major vessel and high density of angiogenesis microvasculature within the tumor. Fig. 12c identifies that major vessel as predominantly hypoxic, and that practically the entire mass is hypoxic. The mass was biopsied and the histology was grade III invasive ductal carcinoma.

The functional parameters provide strong information suspicious of malignancy. Therefore, optoacoustic functional images increase confidence of radiologist interpretation compared to that based on conventional ultrasonic morphological images. Biopsy confirmed invasive breast carcinoma. Fig. 12d–f display another example in which the confidence of the interpreting radiologist of diagnosing breast malignancy increases based on the optoacoustic imaging. The mass is irregularly shaped with indistinct margins on conventional ultrasound, suspicious for malignancy. The gray scale anatomical image level of suspicion for malignancy is not only confirmed, but elevated by the functional images of [tHb] and [sO2]. Biopsy results again showed invasive ductolobular carcinoma grade II.

Fig. 13 gives two examples of how optoacoustic functional images enhance specificity of radiologist interpretation of benign breast fibroadenomas. Fig. 13a depicts an indeterminate lobulated hypoechoic solid breast mass by conventional ultrasound, mildly suspicious for breast cancer (Bi-RADS 4a). Optoacoustic imaging (Fig. 13b) however shows a lack of angiogenesis microvasculature within the tumor. Fig. 13c identifies that a vessel, likely a vein, is pushed away by the mass and drapes over its left anterior margin. The majority of the mass is normally oxygenated. All functional parameters favor a probably benign mass, such as a fibroadenoma, and the lesion can be downgraded to a BI-RAD 3 lesion (probably benign). Biopsy confirmed a benign fibroadenoma. Fig. 13d–f display another similar example in which the optoacoustic imaging information helped the radiologist to downgrade the Bi-RADS assessment from a low suspicion of cancer (BI-RADS 4a) on (d) gray scale ultrasound to A mass that (e) on OA total hemoglobin map has little internal hemoglobin that is (f) entirely well oxygenated on the OA combine oxygenation-deoxygenation map. This mass was downgraded to BI-RADS 3 and could be followed with imaging surveillance. Biopsy results again confirmed a benign fibroadenoma.

Fig. 14 gives two clinical examples of benign masses and demonstrates how optoacoustic functional images enhance the confidence of radiologist interpretation of probably benign masses based on conventional ultrasound. Fig. 14a depicts the morphological image from conventional breast ultrasound of a well-defined homogeneous mildly hypoechoic solid oval-shaped probably benign (BI-RADS 3) mass. Fig. 14b, the OA total hemoglobin map, shows no hemoglobin within or around the mass, indicating no major vessels in the proximity of the tumor and complete absence of angiogenesis microvasculature within the tumor. Fig. 14c, the OA relative oxygenation deoxygenation map, shows that practically the entire mass lacks evidence of hypoxia. There is only a small draping parallel oriented capsular artery along the right anterior margin of the mass. All functional parameters provide a strong evidence to support the benign nature of this mass. Biopsy confirmed a fibroadenoma. Fig. 14d–f displays another similar example of a well-circumscribed mass with low concentration of microvasculature and normal blood oxygenation within the mass. The radiological interpretation of a probably benign mass based on the anatomical image is confirmed by OA and supported by the functional images of [tHb] and [sO2]. Biopsy showed a benign fibroadenoma.

Fig. 15 gives two examples of probably the most useful diagnostic capability of the OA/US dual modality. These images demonstrate how optoacoustic functional imaging can increase suspicion of malignancy from a breast masses with morphology that appears benign on breast ultrasound. Fig. 15a depicts morphological image from conventional breast ultrasound of an oval well-circumscribed hypoechoic mass in the right breast. These imaging features in isolation favor a probably benign lesion such as a fibroadenoma [63,64]. However, Fig. 15b (OA total hemoglobin map) shows significantly increased hemoglobin and vasculature within the mass. Fig. 15c demonstrated that the vasculature in the mass is red - markedly hypoxic. All functional imaging parameters are suspicious for breast carcinoma. Biopsy performed after the diagnostic imaging confirmed grade III invasive ductal carcinoma completely replacing and intramammary lymph node. Fig. 15d–f display another similar patient in which the interpretation changed from a probably benign mass to a mass suspicious for breast carcinoma on the basis of the functional images of [tHb] and [sO2] provided by the Imagio™ system. Once again there is markedly increased total hemoglobin (yellow) and vasculature (e) that is intensely deoxygenated (red) (f) within the mass. Biopsy results confirmed grade III invasive ductal carcinoma.

Fig. 16 provides another type of example when functional images upgrade radiologist level of suspicion at time of ultrasound evaluation from BI-RADS 2 or 3 to BI-RADS 5. Conventional ultrasound image (a) shows a round hypoechoic mass oval-shaped mass. However, the optoacoustic functional images of [tHb] and [sO2] (Fig. 16b,c) demonstrate radially oriented feeding vessels in the tissues that surround the mass (peripheral zone, b and c) as well as intensely deoxygenated partial marginal blush (pink, c). The blush was shown with histologic correlation in this case to be created by high numbers of closely packed vessels, each too small to be resolved individually on OA.

The tumor does not have a well-developed internal angiogenesis, but both the partial marginal/boundary zone deoxygenated blush and the presence of multiple radiating vessel within the peripheral zone have very high positive predictive values for malignancy in the range of 90+%. Optoacoustic imaging provided meaningful information for the radiologist to accurately diagnose malignancy.

## 4 Discussion

The main advantages of optoacoustic imaging are its capabilities of displaying the presence or absence of tumor neovessels and their anatomy as well as displaying functional blood distribution and oxygen saturation properties based on optical absorption differences of hemoglobin and oxyhemoglobin at different laser wavelengths [65]. The development of optoacoustic imaging technology has been spearheaded by its application in the diagnostic imaging of breast masses [66]. The attraction of the optoacoustic imaging systems to radiologists is that it is a non-invasive imaging modality that can be used to diagnose sub-centimeter hyper-vascular masses in the breast without the risk of ionizing x-ray radiation exposure or the need for intravenous contrast or radionuclide injection. In the last 10 years, clinical optoacoustic imaging systems for the detection and/or diagnosis of breast cancer have been actively developed and optoacoustic images compared and correlated with other modalities, such as (x-ray) mammography, magnetic resonance imaging and ultrasound [[67], [68], [69], [70], [71], [72], [73], [74], [75], [76]]. The OA/US imaging system described in this report represents the first optoacoustic technology that demonstrated its clinical relevance for enhanced diagnostic specificity of breast cancer. The main advancements in the system technical specifications and, thus, in clinical performance, were made possible through achieving sufficient sensitivity and the noise equivalent pressure within ultrawide band of ultrasonic frequencies from 0.1 MHz to 10 MHz, and by removal of image artefacts that appear in the hand-held probe, which in turn helped to achieve high volumetric tissue contrast to the depth of up to 40 mm. Furthermore, a lexicon and scoring system was developed for describing the imaging features of the benign and malignant breast lesions as visualized on optoacoustic functional images co-registered with lesion anatomy from conventional ultrasound.

In summary, we developed and described a method of qualitative functional imaging and demonstrated clinical utility of functional parameters such as [tHb] and [sO2] displayed within morphological tissues structures in the breast. Addition of these functional images to the co-registered breast ultrasound images may have potentially transformative impact on breast cancer care in terms of accuracy of diagnosis and differentiation of malignant lesions. Other advantages of Imagio as a clinical modality that require a special report include relative image contrast independence on the age of a patient as well as equal system performance in all racial and ethnical groups.

## 5 Conclusions

By combining optoacoustic (OA) and ultrasonic (US) imaging capability in one system (OA/US), the first clinical modality was developed for functional imaging within specific morphological structures of the breast and thereby satisfied a long-standing need for a more accurate diagnostic imaging of breast cancer. The advanced system design features that enabled contrast and accuracy of functional images co-registered with anatomical images are: (i) the hand-held duplex probe containing an 128 element linear array transducer of ultrawide-band that is protected from interference of both the direct illumination by the light diffusely scattered from tissue back to the probe and the light leaking from the optical fibers inside the probe housing; (ii) sensitive, low noise electronics; (iii) the dual wavelength laser rapidly emitting near-infrared pulses one after the other targeting hemoglobin and oxyhemoglobin; (iv) software with sophisticated signal processing and image reconstruction algorithms enabling real time rate co-registered optoacoustic and ultrasound imaging. Clinical viability of this new technology was demonstrated initially in a feasibility study and then in multicenter clinical trials involving over 2000 patients recruited in 16 leading university hospitals and private diagnostic centers of the United States. The examples of the clinical results presented in this report show that optoacoustic functional images has the potential to provide clinically valuable enhancement of the diagnostic specificity of conventional breast ultrasound by either upgrading morphologically benign masses to cancer (and thus saving lives) or downgrading morphologically suspicious lesions to definitely benign masses (thus alleviating anxiety and saving the costs and complications from unnecessary biopsies) or simply confirming and/or elevating radiologist confidence during interpretation of breast masses based on morphology. Further technical advances of the technology described here are envisioned in the direction of more quantitatively accurate full view three-dimensional optoacoustic tomography systems. These advances could help to enable automated screening in addition to improved diagnostics, especially valuable to replace sensitive but not specific detection by MRI for patients with familial history of breast cancer and simultaneously dense heterogeneous breast [77].

## Conflicts of interest

Authors - technology developers (AAO, JZ, BC, ATS) disclose employment or equity position at Seno Medical Instruments, however, no apparent conflict of interests, and authors - clinical collaborators (WTY, JP), declare no conflicts of interest.

## References

[1] Rosenberg R.D., Hunt W.C., Williamson M.R., Gilliland F.D., Wiest P.W., Kelsey C.A., Key C.R., Linver M.N. (1998). Effects of age, breast density, ethnicity, and estrogen replacement therapy on screening mammographic sensitivity and cancer stage at diagnosis: review of 183,134 screening mammograms in Albuquerque, New Mexico. Radiology.

[2] Oeffinger K.C., Fontham E.T.H., Etzioni R., Herzig A., Michaelson J.S., Shih Y.T., Walter L.C., Church T.R., Flowers C.R., LaMonte S.J., Wolf A.M.D., DeSantis C., Lortet-Tieulent J., Andrews K., Manassaram-Baptiste D., Saslow D., Smith R.A., Brawley O.W., Wender R. (2015). Breast cancer screening for women at average risk. JAMA.

[3] Carney P.A., Miglioretti D.L., Yankaskas B.C., Kerlikowske K., Rosenberg R., Rutter C.M., Geller B.M., Abraham L.A., Taplin S.H., Dignan M., Cutter G., Ballard-Barbash R. (2003). Individual and combined effects of age, breast density, and hormone replacement therapy use on the accuracy of screening mammography. Ann. Intern. Med..

[4] Hubbard R.A., Kerlikowske K., Flowers C.I., Yankaskas B.C., Zhu W., Miglioretti D.L. (2011). Cumulative probability of false-positive recall or biopsy recommendation after 10 years of screening mammography: a cohort study. Ann. Intern. Med..

[5] Gartlehner G., Thaler K.J., Chapman A., Kaminski A., Berzaczy D., Noord M.G.Van, Helbich T.H. (2013). Adjunct ultrasonography for breast cancer screening in women at average risk: a systematic review. Int. J. Evid. Based Healthc..

[6] Vlahiotis A., Griffin B., Stavros A.T., Margolis J. (2018). Analysis of utilization patterns and associated costs of the breast imaging and diagnostic procedures after screening mammography. ClinicoEcon. Outcomes Res..

[7] Berg W.A., Blume J.D., Cormack J.B., Mendelson E.B., Lehrer D., Böhm-Vélez Marcela, Pisano E.D., Jong R.A., Evans W.P., Morton M.J., Mahoney M.C., Hovanessian Larsen L., Barr R.G., Farria D.M., Marques H.S., Boparai K. (2008). And the ACRIN 6666 investigators: combined screening with ultrasound and mammography compared to mammography alone in women at elevated risk of breast Cancer: results of the first-year screen in ACRIN 6666. JAMA.

[8] Neuschler E.I., Butler R., Young C.A., Barke L.D., Bertrand M.L., Böhm-Vélez M., Destounis S., Donlan P., Grobmyer S.R., Katzen J., Kist K.A., Lavin P.T., Makariou E.V., Parris T.M., Schilling K.J., Tucker F.L., Dogan B.E. (2017). Imaging to diagnose benign and malignant breast masses: a new evaluation tool for radiologists. Radiology.

[9] van de Ven S.M.W.Y., Elias S.G., van den Bosch M.A.A.J., Luijten P., Mali W.P.Th.M. (2008). Optical imaging of the breast. Cancer Imaging.

[10] Kruger R.A., Liu P. (1994). Proc. SPIE.

[11] Oraevsky A.A., Jacques S.L., Esenaliev R.O., Tittel F.K. (1994). Laser Based Optoacoustic Imaging in Biological Tissues. Proc. SPIE.

[12] Oraevsky A.A., Vo-Dinh T. (2014). “Optoacoustic tomography: from fundamentals to diagnostic imaging of breast cancer”.

[13] Peters V.G., Wyman D.R., Patterson M.S., Frank G.L. (1990). Optical properties of normal and diseased human breast tissues in the visible and near infrared. Phys. Med. Biol..

[14] Herman G.T. (2010). Fundamentals of Computerized Tomography: Image Reconstruction From Projections.

[15] Emelianov S., Aglyamov S., Shah J., Sethuraman S., Scott G., Schmitt R., Karpiouk A., Motamedi M., Oraevsky A.A. (2004). Synergy of ultrasound, elasticity, and optoacoustic imaging for improved detection and differentiation of cancerous tissue. J. Acoust. Soc. Am..

[16] Niederhauser J.J., Jaeger M., Lemor R., Weber P., Frenz M. (2005). Combined ultrasound and optoacoustic system for real-time high-contrast vascular imaging *in vivo*. IEEE Trans. Med. Imaging.

[17] Garcia-Uribe A., Erpelding T.N., Krumholz A., Ke H., Maslov K., Appleton C., Margenthaler J.A., Wang L.V. (2015). Dual-modality photoacoustic and ultrasound imaging system for noninvasive sentinel lymph node detection in patients with breast cancer. Sci. Rep..

[18] Kim J., Park S., Jung Y., Chang S., Park J., Zhang Y., Lovell J.F., Kim C. (2016). Programmable real-time clinical photoacoustic and ultrasound imaging system. Sci. Rep..

[19] Wang X., Fowlkes J.B., Cannata J.M., Hu C., Carson P.L. (2011). Photoacoustic imaging with a commercial ultrasound system and a custom probe. Ultrasound Med. Biol..

[20] Sivasubramanian K., Periyasamy V., Pramanik M. (2018). Non-invasive sentinel lymph node mapping and needle guidance using clinical handheld photoacoustic imaging system in small animal. J. Biophoton..

[21] Daoudi K., van den Berg P.J., Rabot O., Kohl A., Tisserand S., Brands P., Steenbergen W. (2014). Handheld probe integrating laser diode and ultrasound transducer array for ultrasound/photoacoustic dual modality imaging. Opt. Express.

[22] Folkman J. (1995). Clinical applications of research on angiogenesis. New Engl. J. Med..

[23] Weidner N., Semple J.P., Welch W.R., Folkman J. (1991). Tumor angiogenesis and metastasis - correlation in invasive breast carcinoma. New Engl. J. Med.

[24] Zhu Q., Huang M., Chen N., Zarfos K., Jagjivan B., Kane M., Hedge P., Kurtzman S.H. (2003). Ultrasound-guided optical tomographic imaging of malignant and benign breast lesions: initial clinical results of 19 cases. Neoplasia.

[25] Hsiang D., Shah N., Yu H., Su M.Y., Cerussi A., Butler J., Baick C., Mehta R., Nalcioglu O., Tromberg B. (2005). Coregistration of dynamic contrast enhanced MRI and broadband diffuse optical spectroscopy for characterizing breast cancer. Technol. Cancer Res. Treat..

[26] Chance B., Nioka S., Zhang J., Conant E.F., Hwang E., Briest S., Orel S.G., Schnall M.D., Czerniecki B.J. (2005). Breast cancer detection based on incremental biochemical and physiological properties of breast cancers: a six-year, two-site study. Acad. Radiol..

[27] Cox B., Laufer J.G., Arridge S.R., Beard P.C. (2012). Quantitative spectroscopic photoacoustic imaging: a review. J. Biomed. Opt..

[28] Roggan A., Friebel M., Rschel K Do, Hahn A., Muller G. (1999). Optical properties of circulating human blood in the wavelength range 400–2500 nm. J. Biomed. Opt..

[29] Villringer A., Chance B. (1997). Non-invasive optical spectroscopy and imaging of human brain function. Trends Neurosci..

[30] Wang X., Pang Y., Ku G., Xie X., Stoica G., Wang L.V. (2003). Noninvasive laser-induced photoacoustic tomography for structural and functional in vivo imaging of the brain. Nat. Biotechnol..

[31] Yang J.-M., Favazza C., Chen R., Yao J., Cai X., Maslov K., Zhou Q., Shung K.K., Wang L.V. (2012). Simultaneous functional photoacoustic and ultrasonic endoscopy of internal organs in vivo. Nat. Med..

[32] Dean-Ben X.L., Razansky D. (2013). Functional optoacoustic human angiography with handheld video rate three dimensional scanner. Photoacoustics.

[33] Ermilov S., Fronheiser M., Brecht H.-P., Su R., Conjusteau A., Mehta K., Otto P., Oraevsky A. (2009). Development of laser optoacoustic and ultrasonic imaging system for breast cancer utilizing handheld array probes. Proc. SPIE.

[34] Wang K., Sidky E.Y., Anastasio M.A., Oraevsky A.A., Pan X. (2011). Limited data image reconstruction in optoacoustic tomography by constrained, total variation minimization. Proc. SPIE.

[35] Ermilov S.A., Fronheiser M.P., Nadvoretsky V., Brecht H.-P., Su R., Conjusteau A., Mehta K., Otto P., Oraevsky A.A. (2010). Real-time optoacoustic imaging of breast cancer using an interleaved two laser imaging system coregistered with ultrasound. Proc. SPIE.

[36] Zalev J., Herzog D., Clingman B., Miller T., Kist K., Dornbluth N.C., McCorvey B.M., Otto P., Ermilov S.A., Nadvoretsky V., Conjusteau A., Su R., Tsyboulski D., Oraevsky A.A. (2012). Clinical feasibility study of combined optoacoustic and ultrasonic imaging modality providing coregistered functional and anatomical maps of breast tumors. Proc. SPIE.

[37] A.A. Oraevsky, S.A. Ermilov, A. Conjusteau, P. Brecht, V.V. Nadvoretrskiy, R. Su, D. Herzog, B. Clingman, J. Zalev “Optoacoustic Imaging System Having Handheld Probe Utilizing Optically Reflective Material”, U.S. Patent Application Serial No. US20140039293A1, PCT 13/746,559.

[38] Laser Institute of America, American National Standards for Safe Use of Lasers, ANSI Z136.1 (2014).

[39] Sivasubramanian K., Periyasamy V., Wen K.K., Pramanik M. (2017). Optimizing light delivery through fiber bundle in photoacoustic imaging with clinical ultrasound system: Monte Carlo simulation and experimental validation. J. Biomed. Opt..

[40] Spirou G.M., Oraevsky A.A., Vitkin I.A., Whelan W.M. (2005). Optical and acoustic properties at 1064 nm of Polyvinyl-chloride-plastisol for use as a tissue phantom in biomedical optoacoustics. Phys. Med. Biol..

[41] Vogt W.C., Jia C., Wear K.A., Garra B.S., Pfefer T.J. (2016). Biologically relevant photoacoustic imaging phantoms with tunable optical and acoustic properties. J. Biomed. Opt..

[42] Morse P.M., Ingard K.U. (1968). Theoretical Acoustics.

[43] Kino G.S. (1987). Acoustic Waves, Devices, Imaging and Analog Signal Processing.

[44] Karabutov A.A., Savateeva E.V., Podymova N.B., Oraevsky A.A. (2000). Backward detection of laser-induced wide-band ultrasonic transients with optoacoustic transducer. J. Appl. Phys..

[45] Ermilov S.A., Fronheiser M.P., Brecht H.-P., Su R., Conjusteau A., Mehta K., Otto P., Oraevsky A.A. (2009). Development of laser optoacoustic and ultrasonic imaging system for breast cancer utilizing hand-held array probe. Proc. SPIE.

[46] Wang K., Ermilov S.A., Su R., Brecht H.-P., Anastasio M.A., Oraevsky A.A. (2011). An imaging model incorporating ultrasonic transducer properties for three-dimensional optoacoustic tomography. IEEE Trans. Med. Imaging.

[47] Kruger R.A., Liu P., Fang Y.R., Appledorn C.R. (1995). Photoacoustic ultrasound (PAUS) reconstruction tomography. Med. Phys..

[48] Oraevsky A.A., Andreev V.G., Karabutov A.A., Esenaliev R.O. (1999). Two-dimensional optoacoustic tomography: array transducers and image reconstruction algorithm. Proc. SPIE.

[49] Xu M., Wang L.V. (2005). Universal back-projection algorithm for photoacoustic computed tomography. Phys. Rev..

[50] Wang K., Anastasio M.A., Scherzer Otmar (2011). Photoacoustic and thermoacoustic tomography: image formation principles. Handbook of Mathematical Methods in Imaging.

[51] van Veen R.L.P., Sterenborg H.J.C.M., Marinelli A.W.K.S., Menke-Pluymers M. (2004). Intraoperatively assessed optical properties of malignant and healthy breast tissue used to determine the optimum wavelength of contrast for optical mammography. J. Biomed. Opt..

[52] Cerussi A., Shah N., Hsiang D., Durkin A., Butler J., Tromberg B.J. (2006). In vivo absorption, scattering, and physiologic properties of 58 malignant breast tumors determined by broadband diffuse optical spectroscopy. J. Biomed. Opt..

[53] Grosenick D., Wabnitz H., Moesta K.T., Mucke J., Schlag P.M., Rinneberg H. (2005). Time-domain scanning optical mammography: II. Optical properties and tissue parameters of 87 carcinomas. Phys. Med. Biol..

[54] Pogue B.W., Poplack S.P., McBride T.O., Wells W.A., Osterman K.S., Osterberg U.L., Paulsen K.D. (2001). Quantitative hemoglobin tomography with diffuse near-infrared spectroscopy: pilot results in the breast. Radiology.

[55] Ghosh N., Mohanty S.K., Majumder S.K., Gupta P.K. (2001). Measurement of optical transport properties of normal and malignant human breast tissue. Appl. Opt..

[56] Spinelli L., Torricelli A., Pifferi A., Taroni P., Danesini G.M., Cubeddu R. (2004). Bulk optical properties and tissue components in the female breast from multiwavelength time-resolved optical mammography. J. Biomed. Opt..

[57] D’astous F.T., Foster F.S. (1986). Frequency dependence of ultrasound attenuation and backscatter in breast tissue. Ultrasound Med. Biol..

[58] Geise R.A., Palchevsky A. (1996). Composition of mammographic phantom materials. Radiology.

[59] J. Zalev, B. Clingman: "Statistical mapping in an optoacoustic imaging system." U.S. Patent 9,836,838, December 5, 2017.

[60] Wanders J.O.P., Holland K., Veldhuis W.B., Mann R.M., Pijnappel R.M., Peeters P.H.M., van Gils C.H., Karssemeijer N. (2017). Volumetric breast density affects performance of digital screening mammography. Breast Cancer Res. Treat..

[61] Kerlikowske K., Scott C.G., Mahmoudzadeh A.P., Ma L., Winham S., Jensen M.R., Wu F.F., Malkov S., Pankratz V.S., Cummings S.R., Shepherd J.A., Brandt K.R., Miglioretti D.L., Vachon C.M. (2018). Automated and clinical breast imaging reporting and data system density measures predict risk of screen-detected and interval cancers. Ann. Intern. Med..

[62] Liao D., Johnson R.S. (2007). Hypoxia: a key regulator of angiogenesis in cancer. Cancer Metastasis Rev..

[63] American College of Radiology. American College of Radiology, Breast Imaging Reporting and Data System Atlas (BI-RADS Atlas). Vol. 5. Reston, VA: American College of Radiology; 2013.

[64] Stavros A.T. (2004). Breast Ultrasound.

[65] A.A. Oraevsky, A.A. Karabutov Optoacoustic Tomography, Biomedical Photonics Handbook, T. Vo-Dinh, CRC Press, Boca Raton, Florida, 2003, Vol. PM125, Chapter 34, pp. 34/1-34/34.

[66] Oraevsky A.A., Andreev V.G., Karabutov A.A., Fleming D.R., Gatalica Z., Sindh H., Esenaliev R.O. (1999). Laser optoacoustic imaging of the breast: detection of cancer angiogenesis. Proc. SPIE.

[67] Ermilov S.A., Khamapirad T., Conjusteau A., Lacewell R., Mehta K., Miller T., Leonard M.H., Oraevsky A.A. (2009). Laser optoacoustic imaging system for detection of breast cancer. J. Biomed. Opt..

[68] Heijblom M., Piras D., Xia W., van Hespen J.C., Klaase J.M., van den Engh F.M., van Leeuwen T.G., Steenbergen W., Manohar S. (2012). Visualizing breast cancer using the Twente photoacoustic mammoscope: what do we learn from twelve new patient measurements?. Opt. Express.

[69] Kitai T., Torii M., Sugie T., Kanao S., Mikami Y., Shiina Ti, Toi M. (2012). Photoacoustic mammography: initial clinical results. Breast Cancer.

[70] Kruger R.A., Kuzmiak C.M., Lam R.B., Reinecke D.R., Del Rio S.P., Steed D. (2013). Dedicated 3D photoacoustic breast imaging. Med. Phys..

[71] Fakhrejahani E., Torii M., Kitai T., Kanao S., Asao Y., Hashizume Y., Mikami Y., Yamaga I., Kataoka M., Sugie T., Takada M., Haga H., Togashi K., Shiina T., Toi M. (2015). Clinical report on the first prototype of a photoacoustic tomography system with dual illumination for breast cancer imaging. PLoS One.

[72] Heijblom M., Piras D., van den Engh F.M., van der Schaaf M., Klaase J.M., Steenbergen W., Manohar S. (2016). The state of the art in breast imaging using the Twente photoacoustic mammoscope: results from 31 measurements on malignancies. Eur. Radiol..

[73] Valluru Keerthi S., Wilson Katheryne E., Willmann Jürgen K. (2016). Photoacoustic imaging in oncology: translational preclinical and early clinical experience. Radiology.

[74] Toi M., Asao Y., Matsumoto Y., Sekiguchi H., Yoshikawa A., Takada M., Kataoka M., Endo T., Kawaguchi-Sakita N., Kawashima M., Fakhrejahani E., Kanao S., Yamaga I., Nakayama Y., Tokiwa M., Torii M., Yagi T., Sakurai T., Togashi K., Shiina T. (2017). Visualization of tumor-related blood vessels in human breast by photoacoustic imaging system with a hemispherical detector array. Sci. Rep..

[75] Deán-Ben X.L., Fehm T.F., Gostic M., Razansky D. (2016). Volumetric hand-held optoacoustic angiography as a tool for real-time screening of dense breast. J. Biophoton..

[76] Diot G., Metz S., Noske A., Liapis E., Schroeder B., Ovsepian S.V., Meier R., Rummeny E., Ntziachristos Vasilis (2018). Multispectral optoacoustic tomography (MSOT) of human breast Cancer. Clin. Cancer Res..

[77] Kuhl C.K., Schrading S., Leutner C.C., Morakkabati-Spitz N., Wardelmann E., Fimmers R., Kuhn W., Schild H.H. (2005). Mammography, breast ultrasound, and magnetic resonance imaging for surveillance of women at high familial risk for breast cancer. J. Clin. Oncol..

